# Morpho-Physiological and Biochemical Responses of Hydroponically Grown Basil Cultivars to Salt Stress

**DOI:** 10.3390/antiox11112207

**Published:** 2022-11-08

**Authors:** Michele Ciriello, Luigi Formisano, Marios C. Kyriacou, Petronia Carillo, Luca Scognamiglio, Stefania De Pascale, Youssef Rouphael

**Affiliations:** 1 Department of Agricultural Sciences, University of Naples Federico II, 80055 Portici, Italy; 2 Department of Vegetable Crops, Agricultural Research Institute, 1516 Nicosia, Cyprus; 3 Department of Environmental, Biological and Pharmaceutical Sciences and Technologies, University of Campania “Luigi Vanvitelli”, 81100 Caserta, Italy

**Keywords:** antioxidant capacity, bioactive molecules, carotenoids, flavonoids, osmotic stress, phenolic acids, secondary metabolites

## Abstract

Depending on duration and magnitude, abiotic stresses interfere with plant metabolic processes and may severely impact developmental and qualitative attributes. In this study, in addition to characterizing three different cultivars of basil (‘Anise’, ‘Cinnamon’, and ‘Lemon’) grown under hydroponics, we appraised the impact of NaCl salt stress (60 mM) on morphophysiological and nutraceutical properties of the basil crop. Salt stress significantly reduced fresh yield (51.54%, on average) and photosynthetic parameters (ACO_2_, E, and gs) in all cultivars by raising tissue concentrations of Na^+^ and Cl^−^. In addition to reducing the concentration of nitrate (77.21%), NaCl salt stress increased the concentrations of key bioactive molecules, notably carotenoids (lutein and *β*-carotene), phenolic acids, and flavonoid derivatives, thus resulting in a higher antioxidant activity of salt-treated basil plants compared to the untreated ones. Analysis by UHPLC revealed that cichoric acid was the most abundant polyphenolic compound in all basil cultivars, with the highest values recorded in ‘Cinnamon’.

## 1. Introduction

The world population has peaked at eight billion people, and recent estimates project an increase of more than two billion people by 2050. In the coming decades, the imperative for community policies will be to ensure food security, a daunting challenge when we consider that it is mainly dependent on agricultural production [1]. Currently, the agricultural sector is managing to bridge the gap between demand and production, but demographic growth will put further pressure on the entire sector. As if that were not enough, changing climate scenarios have thrown even more fuel on the fire, endangering the food supply for future generations.

The increase in the frequency and intensity of drought has led to an increase in desertification with consequent soil salinization, a concern that should not be underestimated considering that horticultural crops are widespread in regions with high levels of water salinity [2,3,4]. In an era of climate change, salinity is undoubtedly a constraining factor in agricultural cultivation [5]. More than 20% of irrigated agricultural soils have high levels of salinity, resulting from the natural erosive process (i.e., primary salinity) but mainly from anthropogenic activities (i.e., secondary salinity) such as intensifying agricultural practices, deforestation, and irrigation, promoting seawater infiltration [6]. If not remedied, negative salinity impacts will affect 50% of the world’s agricultural land by 2050. NaCl salinity causes a rapid osmotic effect that has drastic consequences on plant water and nutrient availability, photosynthetic and transpiration rates, stomatal regulation and control mechanisms, and root functional activities [7,8,9,10,11]. If the stress continues, morphological and physiological changes could occur, leading to reduced yields. Taking into account the long-term economic unsustainability of water and soil desalination processes and the low availability of salt-tolerant genotypes, finding an appropriate use for salinized areas is one of the biggest challenges. One possible solution could be to allocate such regions to the cultivation of medicinal plants, appreciated in gastronomic, pharmaceutical, medical, and cosmetic fields due to their richness in secondary metabolites. In fact, although salt stress leads to the production of reactive oxygen species (ROS), which limits production performance, it prompts the plant to activate defensive mechanisms that culminate in the bioaccumulation of phenolic molecules with an antioxidant function. As observed by Valifard et al. [12] on *Salvia mirzayanii* and *Salvia acrosiphon* and by Perin et al. [13] on *Fragaria ananassa*, the increase in polyphenols in response to salt stress is positively related to the up-regulation of phenylalanine ammonia-lyase (PAL) enzyme activity. Although secondary metabolites do not play a specific role in growth processes in medicinal plants, these valuable species-specific molecules increase under stress conditions, improving plant quality traits. The growing interest in products of natural origin that are readily available and have no side effects has increased the demand for medicinal plants because of their beneficial antimicrobial and anti-inflammatory properties, making them inexpensive and renewable “ingredients” for producing natural preservatives, new types of drugs, and cosmetics [14,15].

Currently, in developing countries, about 80% of medicines are of plant origin, while in developed countries, the proportion is only 25%. Among medicinal plants, basil (*Ocimum basilicum* L.), which is exceptionally rich in essential oils, is among the most widespread and well known worldwide, so much so that it has earned the nickname “King of Herbs”, with the existence of at least 18 cultivars selected and developed over the years [16]. Its fame is mainly attributable to its gastronomic role as tender and fragrant leaves, critical ingredients of tasty dishes typical of Italian gastronomic tradition [17]. Not surprisingly, as with other plants belonging to the Lamiaceae family, basil, in addition to its recognized culinary aptitude, is cultivated for its secondary metabolites. Most studies have focused primarily on Genovese basil’s productive and sensory characteristics without considering the genetic diversity typical of the genus Ocimum. Over time, many basil cultivars that have genetically distinct phytochemical profiles have been selected for their shape, color, aroma, and flavor. Basil contains relatively high concentrations of carotenoids (lutein and *β*-carotene) and polyphenols, which belong mainly to flavonol-glycoside classes (rutin, quercetin, and di-hydroquercetin) and phenolic acids (rosmarinic, chicoric, caffeic, chlorogenic, kaftaric, salvianic A, salvianolinic A, L and K acids), which, in addition to acting as stress mitigators, are beneficial for human health. However, although Genovese basil cultivated in soil is known to be tolerant of salinity (up to 100 mM NaCl) under, few studies have evaluated the effects of salt stress on the polyphenolic profile of non-Genovese basil for the pharmaceutical and cosmetic industries. The few contributions available in the literature have mainly focused on evaluating the effects of salinity on the yield and quality of Genovese basil while neglecting other non-Genovese types belonging to the genus Ocimum. Our work was aimed at characterizing three types of basil (*Ocimium basilicum* var thyrsiflora, *Ocimum basilicum* cv Cinnamon, and *Ocimum* × *Citriodorum*) under salt stress, both from a yield and phytochemical point of view. We evaluated the yield, morphophysiological response, antioxidant activity [FRAP (ferric ion reducing antioxidant power), DPPH (1,1-diphenyl-2-picrylhydrazyl), and ABTS (2,2′-azinobis-(3-ethylbenzothiazoline-6-sulfonate)], mineral profile (by ion chromatography) and phenolic profile (by ultrahigh performance liquid chromatography).

## 2. Materials and Methods

### 2.1. Experimental Site and Plant Material

**Figure 1:**
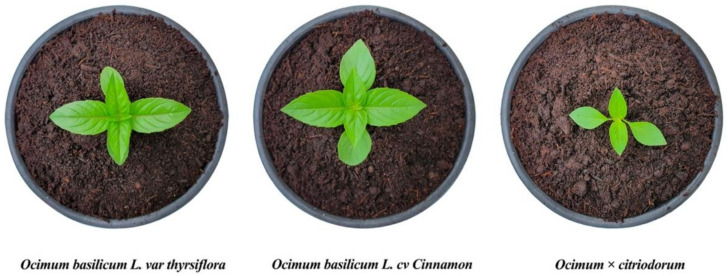
Illustrative pictures of the different types of basil at transplant. From left to right: *Ocimum basilicum* L. var thyrsiflora (Anise), *Ocimum basilicum* L. cv Cinnamon, and *Ocimum × citriodorum* (Lemon).

The experimental trial, aimed at evaluating the effects of NaCl salt stress in basil (*Ocimum basilicum* L.), was carried out in spring–summer 2021 in the greenhouses of the Federico II University of Naples Department of Agriculture (DIA) (Portici, Italy; 40°48′ N, 14°20′ E, 29 m.s.l.). On 5 May 2021, basil plants ‘Anise’ (*Ocimum basilicum* L. var thyrsiflora; Blumen, Milan, Italy), ‘Cinnamon’ (*Ocimum basilicum* L. cv Cinnamon; Blumen, Milan, Italy) and ‘Lemon’ (*Ocimum × citriodorum*; Pagano Domenico & Figli Sementi, Scafati, Italy) were seeded in 54-hole polystyrene trays (52 × 32 × 6 cm, L × W × D; volume: 0.06 L) until two true leaves appeared. At 14 days after sowing (29 May 2021; 1 day after transplanting), seedlings were transplanted into round anti-spiraling pots (0.1 m × 0.1 m × 0.15 m) filled with a mixture (*v/v*) of 1/3 perlite and 2/3 peat (Vigorplant, Fombio, Italy) (Figure 1). The pots were placed in rows with a spacing of 0.27 m × 0.15 m with a density of 25 plants m^−2^. Nutrient solution (NS) was distributed through drippers with a flow rate of 2 L h^−1^ (1 dripper/plant).

### 2.2. Experimental Design

Basil seedlings were arranged in the greenhouse according to a bifactorial design in which three basil cultivars (‘Anise’, ‘Cinnamon’, and ‘Lemon’) and two NSs (salt and a non-salt control) were considered as factors. The control NS was a modified Hoagland with the following nutrient element composition: 13.0 mM NO_3_-N, 1.0 mM NH_4_-N, 1.5 mM P, 5.0 mM K, 1.75 mM S, 4.5 mM Ca, 2 mM Mg, 9 μM Mn, 20 μM Fe, 0.3 μM Cu, 20 μM B, 1.6 μM Zn, and 0.3 μM Mo. Each experimental unit was replicated three times and included 15 plants (45 plants per treatment). Saline NS was prepared by adding 60 mM NaCl to the control NS.

### 2.3. Plant Collection

At harvest (26 days), eight plants per replicate were cut at root collar and sampled to determine biometric parameters and yield. Freshly sampled plants were weighed for total fresh and leaf weight measurements (g plant^−1^). The height (cm) and number of leaves per plant were then determined. The collected samples were dried in a ventilated oven at 60 °C until constant weight (about three days) for the measurement of the dry weight (g plant^−1^) and the percentage of dry matter. The dried plant material was then finely ground using an MF10.1 cutting head mill (IKA^®^, Staufen im Breisgau, Baden-Württemberg, Germany) for the measurement of the mineral concentration. Four plants per replicate were sampled and immersed in liquid nitrogen, stored at −80 °C, and subjected to a freeze-drying cycle (Christ, Alpha 1–4 (Martin Christ Gefriertrocknungsanlagen GmbH, Osterode am Harz, Germany) for the measurement of antioxidant activity, carotenoids, and phenolic acids, while another part was stored at −20 °C for the measurement of chlorophyll concentration.

### 2.4. Digital Quantification of the Leaf Area

Digital quantification of the leaf area was performed using photos of the leaves of each plant sampled. Specifically, at 26 days, the leaves of each plant were placed on a white panel perpendicular to the camera lens (Nikon D80, Nikon AF S DX 18-135/3.5-5.6G IF-ED lens; Nikon Corporation, Tokyo, Japan). The captured photos were processed with Adobe^®^ Lightroom Classic and Adobe^®^ Photoshop 2022 software (Adobe Inc., San Jose, CA, USA) for distortion correction and brightness and contrast adjustment. Leaf area was quantified using ImageJ v1.52a software (U.S. National Institutes of Health of the United States, Bethesda, Rockville, MD, USA). The analyses were performed in triplicate and the leaf area was expressed in cm^2^.

### 2.5. Color Measurement

Just before harvest, colorimetric indices were determined on ten healthy and fully expanded leaves per replicate using a Minolta CR-400 portable colorimeter (Minolta Camera Co. Ltd., Osaka, Japan). Color was converted according to the CIE 1976 L, a*, b* (CIELAB) color space, where L indicates brightness, a and b chromaticity (greenness and yellowness, respectively). Chroma and Hue angle (°) were calculated according to the formulas proposed by Kheng [18].

(1) $$Chroma\;=\;\sqrt{a^{*2}+b^{*2}}$$

(2) $$Hue\;=\;tan^{-1}\left(\frac{a^{*}}{b^{*}}\right)$$

where,

$$0{}^{\circ}<Hue<90{}^{\circ}\;if\;a^{*},b^{*}\;>0$$

$$90{}^{\circ}<Hue<180{}^{\circ}\;if\;a^{*}<0,b^{*}>0$$

$$180{}^{\circ}<Hue<270{}^{\circ}\;if\;a^{*},b^{*}<0$$

$$270{}^{\circ}<Hue<360{}^{\circ}\;if\;a^{*}>0,b^{*}<0$$

### 2.6. Leaf Gas Exchange and Leaf Fluorescence

At 26 days, six fully expanded and well illuminated leaves per replicate were labeled and used to determine gas exchange and leaf fluorescence. Measurements were taken in the middle of the day (11:00 a.m.–3:00 p.m. solar time).

The net CO_2_ assimilation rate (ACO_2_; μmol CO_2_ m^−2^ s^−1^), transpiration (E; mmol H_2_O m^−2^ s^−1^), and stomatal conductance (gs; mol H_2_O m^−2^ s^−1^) were measured with the LI-6400 portable gas exchange system (LI-COR Biosciences, Lincoln, NE, USA). The CO_2_ in the measurement chamber was set at ambient values (about 400 ppm) and the photosynthetically active radiation at 1000 µmol m^−2^ s^−1^. The leaves were closed in the measurement chamber until equilibrium was reached (about 15 min).

Chlorophyll fluorescence measurement was performed using a portable F_v_/F_m_ Meter fluorometer (Plant Stress Kit, Opti-Sciences, Hudson, NH, USA) on dark-adapted leaves for 10 min using leaf clips. Maximum chlorophyll fluorescence (F_m_) was induced with a saturating light pulse of 3000 μmol photons m^−2^ s^−1^ for 1s, while initial fluorescence (F_o_) was recorded with an internal blue LED light of 1–2 μmol photons m^−2^ s^−1^. F_v_/F_m_ was estimated as (F_m_ − F_o_)/F_m_.

### 2.7. Pigment Measurement

The concentrations of leaf chlorophyll and carotenoid (lutein and *β*-carotene) concentrations were determined by spectrophotometry and high-performance liquid chromatography with diode array detection (HPLC-DAD), respectively. All reagents were purchased from Sigma-Aldrich (Milan, Italy). Analyses were performed in triplicate.

To determine chlorophyll according to Wellburn [19]’s protocol, 0.5 g of fresh frozen sample was extracted in 3 mL of 90% ammonia acetone (*v*/*v*) and homogenized by IKA^®^ T10 basic Ultra Turrax^®^ homogenizer (Staufen im Breisgau, Baden-Württemberg, Germany). Chlorophyll a and b concentrations were determined using a UV-Vis spectrophotometer (DR 4000, Hach Co., Loveland, CO, USA) with an absorbance of 647 and 664 nm, respectively. Total chlorophyll was calculated as chlorophyll a + chlorophyll b and expressed as mg g^−1^ of fresh weight (fw).

Lutein and *β*-carotene concentrations were determined in freeze-dried basil leaves according to the protocol of Salomon et al. [20]. A 0.1 g freeze-dried sample was extracted in a mixture of ultra-pure water (1 mL) and ethanol/n-hexane (5 mL; 60:50, *v*/*v*) and subjected to a vacuum centrifugation cycle to separate the pellet from the solvent. The pellet was mixed with methanol and MTBE (methyl-t-butyl ether) in a 1:1 (*v*/*v*) ratio and analyzed by HPLC-DAD. The results were expressed as mg kg^−1^ of dry weight (dw).

### 2.8. Antioxidant Activity

To determine the antioxidant activity, three spectrophotometric methods were compared: DPPH (1,1-diphenyl-2-picrylhydrazyl), ABTS (2,2′-azinobis-(3-ethylbenzothiazoline-6-sulfonate)), and FRAP (ferric ion reducing antioxidant power), according to protocols of Brand-Williams et al. [21], Re et al. [22], and Rajurkar and Hande [23], respectively.

To determine the antioxidant activity of DPPH, 1 mL of DPPH solution (0.1 mM) and 96% methanol were added to 200 μL of the aqueous extract, mixed and incubated at room temperature for 30 min in the dark. The absorbance was recorded against the blank at 517 nm.

To determine ABTS antioxidant activity, a stock solution of ABTS^+^ was prepared by reacting 7 mM aqueous solution of ABTS^+^ with 2.45 mM aqueous solution of potassium persulfate in equal parts. After incubation in the dark (16 h at 23 °C), the stock solution was diluted with ethanol in a ratio of 1:88 until an absorbance of 0.700 ± 0.020 at 734 nm was reached. A 0.1 mL aliquot of each sample, previously filtered and diluted (1:10) with 70% methanol, was mixed with 1 mL of ABTS^+^ solution and stored at room temperature for 2.5 min. The absorbance was read at 734 nm.

For measurement of FRAP antioxidant activity, a FRAP solution was prepared containing 1.25 mL of Fe^2+^/2,4,6-tris (2-pyridyl)-s-triazine (10 mM) in HCl (40 mM) + 1.25 mL of FeCl_3_ (20 mM) in water + 12.5 mL of acetate buffer (0.3 M, pH 3.6). An aliquot of 150 μL of the sample was mixed with 2.850 mL of FRAP solution and incubated for 4 min in the dark. The absorbance was read at 593 nm against a blank containing all the reagents.

The absorbances of the DPPH, ABTS, and FRAP essays were recorded with a UV-vis spectrophotometer (Shimadzu, Japan). The results were expressed as Trolox equivalent mmol kg^−1^ dw. All analyses were performed in triplicate.

### 2.9. Measurement of the Polyphenolic Profile

For the measurement of the phenolic profile, 5 μL samples extracted according to the procedure described by Vallverdu-Queralt et al. [24] were analyzed by ultrahigh performance liquid chromatography (UHPLC; Dionex Ultimate 3000, Thermo Fisher Scientific^TM^, Waltham, MA, USA) and Orbitrap high-resolution mass spectrometry (HRMS; Thermo Fisher Scientific^TM^, Waltham, MA, USA) according to the protocol detailed by El-Nakhel et al. [25]. The chromatographic separation of polyphenols was carried out with a Luna Omega PS column (1.6 μm, 50 × 2.1 mm, Phenomenex, Torrance, CA, USA) set at 25 °C, in which the mobile phases consisted of water (A) and acetonitrile (B), both containing 0.1% formic acid (*v*/*v*). The Q-Exactive Orbitrap mass spectrometer was set in a fast negative/positive ion switching mode with two scan events (Full ion MS and all-ion fragmentation, AIF) for all compounds of interest. The calibration and accuracy of the equipment was monitored using a standard reference mixture (Thermo Fisher Scientific^TM^, Waltham, MA, USA). Data processing was performed with Quan/Qual Browser Xcalibur software, v. 3.1.66.10 (Thermo Fisher Scientific^TM^, Waltham, MA, USA). Polyphenols were expressed as mg kg^−1^ dw.

### 2.10. Mineral Concentration

The measurement of cations (K, Ca, Mg, and Na) and anions (Nitrate, P, and Cl) of basil leaves was carried out by ion chromatography coupled with an electrical conductivity detector (ICS3000, Thermo Scientific^TM^ Dionex^TM^, Sunnyvale, CA, USA) according to the detailed protocol of Formisano et al. [26]. By comparing peak areas, the integration and quantification of mineral concentration was performed using the z Chromeleon^TM^ 6.8 Chromatography Data System software (Thermo Scientific^TM^ Dionex^TM^, Sunnyvale, CA, USA) data of samples with those of certified reference standards.

All treatments were analyzed in triplicate and the concentrations of the concentrations of anion and cations were expressed in g kg^−1^ dw, except for the for the nitrate, expressed in mg kg^−1^ fw.

### 2.11. Statistics

Statistical analysis was performed with IBM SPSS 20 software (Armonk, NY, USA) for Microsoft Windows 11. A two-way analysis of variance (ANOVA) was applied to assess the significance of the effects and interactions between the cultivar factors (CV) and salt stress (S). The mean effect of CV factor was compared by one-way analysis of variance, and the mean effect of S was compared by Student’s *t* test. Statistical significance of the CV × S interaction and the CV factor was determined using the Tukey–Kramer HSD test at the level of *p* < 0.05. All data were presented as mean ± standard error.

## 3. Results

### 3.1. Biometric Parameters

**Figure 2:**
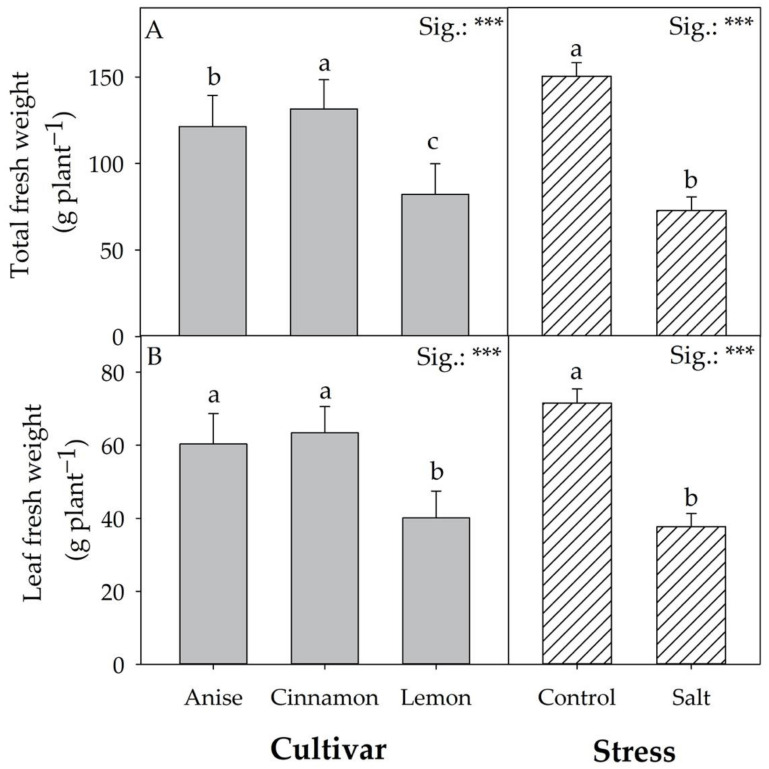
Effect of Cultivar and Stress on total fresh weight (**A**) and leaf fresh weight (**B**). Data are mean values ± standard error, *n* = 3. Mean comparisons were performed by Tukey HSD test for Cultivar and by *t*-Test for Stress. Different letters indicate significant mean differences. *** denotes significant effects at *p* ≤ 0.001.

**Figure 3:**
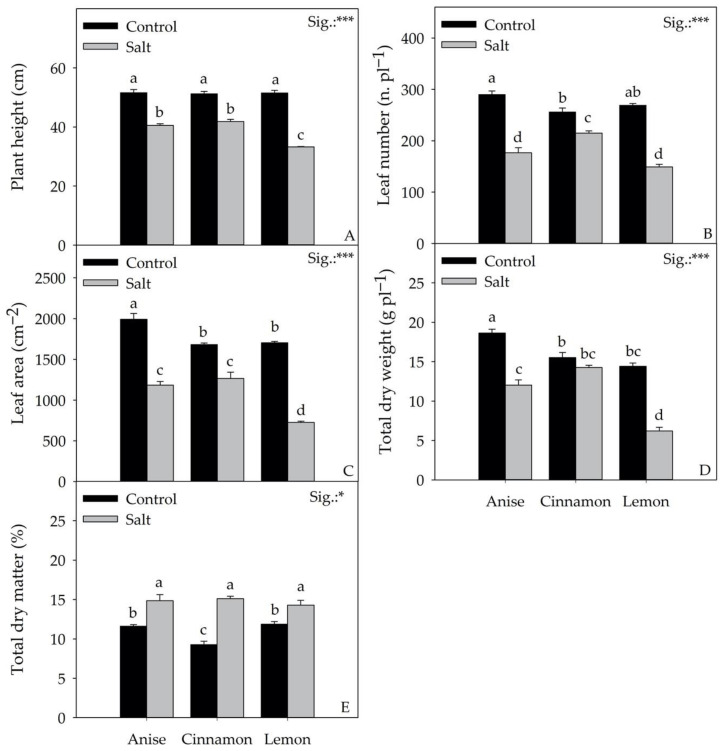
Effect of Cultivar × Stress on plant height (**A**), leaf number (**B**), leaf area (**C**), total dry weight (**D**), total dry matter (**E**). Data are mean values ± standard error, *n* = 3. Statistical significance of the CV × S interaction was determined by Tukey HSD test for Cultivar and by *t*-Test for Stress. Different letters indicate significant mean differences. * and *** denote significant effects at *p* ≤ 0.05 and 0.001, respectively.

Except for total fresh weight and fresh leaf weight (Figure 2A,B), the biometric parameters shown in Figure 3 and Supplementary Table S1 were significantly affected by the interaction between cultivar (CV) and stress (S) factors. Compared to the Control, salt treatment (Salt) reduced the total fresh and leaf weight by 51.52 and 47.32%, regardless of the cultivar (Figure 2A,B). The highest total fresh weight was found in the cultivar Cinnamon, followed by ‘Anise’ and ‘Lemon’. Relative to fresh leaf weight, no significant differences were observed between ‘Cinnamon’ and ‘Anise’, unlike ‘Lemon’, which showed the lowest value (40.11 g plant^−1^). Compared to the Control, Salt treatment reduced the number of leaves by 39.15, 16.11, and 44.54% in ‘Anise’, ‘Cinnamon’, and ‘Lemon’, respectively (Figure 3B). A similar trend was observed for the leaf area, with the lowest value obtained from the Lemon × Salt interaction (725.29 cm^2^; Figure 3C). This latter interaction also resulted in the lowest total dry weight (6.20 g plant^−1^; Figure 3D). Differently, the percentage of dry matter of all cultivars increased significantly after salt treatment.

### 3.2. Colorimetric Indices

As shown in Table 1, the CIELAB colorimetric parameters a*, b*, the chroma and the Hue angle were significantly affected by the interaction of the CV and S factors. Specifically, for ‘Lemon’, an increase in a* (+18.43%) and Hue angle (+2.21%) was observed in plants under salt stress, compared to the Control, while b* (−26.49%) and Chroma (23.79%) decreased. In ‘Anise’ and ‘Cinnamon’, Salt treatment did not result in significant differences for a^*^ and Chroma. An opposite trend was observed for the hue angle between the Anise and Cinnamon cultivars. Specifically, compared with Control, the above colorimetric parameter was not affected by salt treatment in ‘Anise’, while in ‘Cinnamon’ there was a 1.90% increase.

### 3.3. Leaf Physiological Parameters

**Figure 4:**
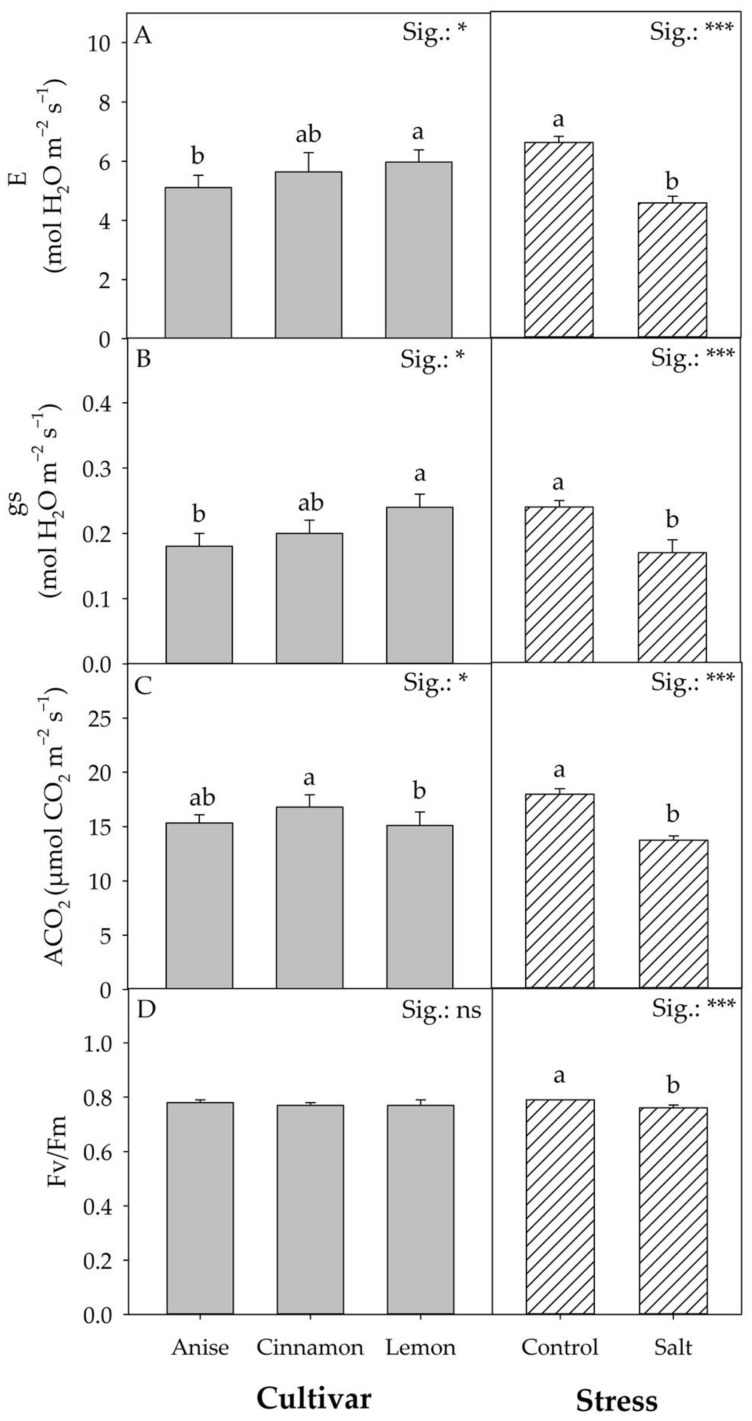
Effect of Cultivar and Stress on transpiration (**A**), stomatal conductance (**B**), net CO_2_ assimilation rate (**C**), and chlorophyll fluorescence (**D**). Data are mean values ± standard error, *n* = 3. Mean comparisons were performed by Tukey HSD test for Cultivar and by *t*-Test for Stress. Different letters indicate significant mean differences. ns, *, and *** denote non-significant or significant effects at *p* ≤ 0.05 and 0.001, respectively.

The CV × S interaction did not result in significant differences for the physiological parameters shown in Figure 4 and Supplementary Table S2. Regardless of the cultivar, compared to the control, salt stress significantly (*p* < 0.001) reduced net CO_2_ assimilation (ACO_2_), transpiration (E), and stomatal conductance (gs) by 23.72, 30.95, and 29.16%, respectively. The same parameters were influenced by the mean effect of the cultivar. Specifically, ‘Lemon’ showed the highest E and gs values compared to ‘Anise’, but the lowest ACO_2_ values compared to ‘Cinnamon’. On the contrary, the maximum quantum efficiency of photosystem II (F_v_/F_m_) was solely affected by S. The data in Figure 4 D show that plants under salt stress decreased the F_v_/F_m_ values (3.79%) compared to the control.

### 3.4. Leaf Pigments

**Figure 5:**
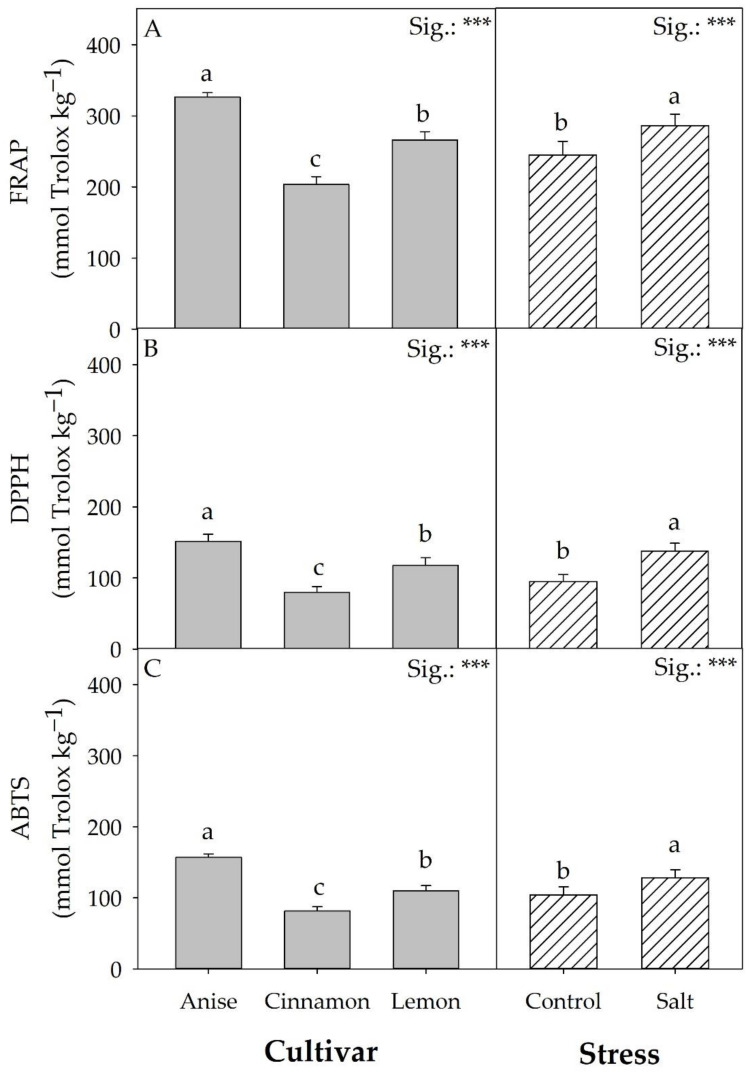
Effect of basil cultivars and stress on FRAP (**A**), DPPH (**B**), and ABTS (**C**) antioxidant activity. Data are mean values ± standard error, *n* = 3. Mean comparisons were performed by Tukey HSD test for CV and by *t*-Test for S. Different letters indicate significant mean differences. *** denotes significant effects at *p* ≤0.001.

Chlorophyll a, b, and total chlorophyll concentrations were not affected by the factors considered nor by their mutual interaction, unlike lutein and *β*-carotene (Table 2). The lutein concentration showed significant differences for the mean CV and S. Specifically, ‘Lemon’ had 25.75% lower lutein concentrations (on average) than the other cultivars. Regardless of the cultivar, salt stress increased the latter carotenoid by 22.85% compared to the control. Compared to the control, the interactions of the ‘Anise’ and ‘Cinnamon’ cultivars with salt treatment (Salt) increased *β*-carotene concentration by 39.17 and 47.67%, respectively. The same trend was also observed for antioxidant activity, where salinity increased by 16.18, 44.96, and 23.28% FRAP, DPPH, and ABTS essays, respectively (Figure 5). It should be noted that regardless of the antioxidant activity assay, the highest concentrations were recorded in ‘Anise’, followed by ‘Lemon’ and ‘Cinnamon’.

### 3.5. Phenolic Acids

Table 3 and Table 4 show the phenolic acid and flavonoid derivatives of basil leaves, respectively, under salt stress.

**Figure 6:**
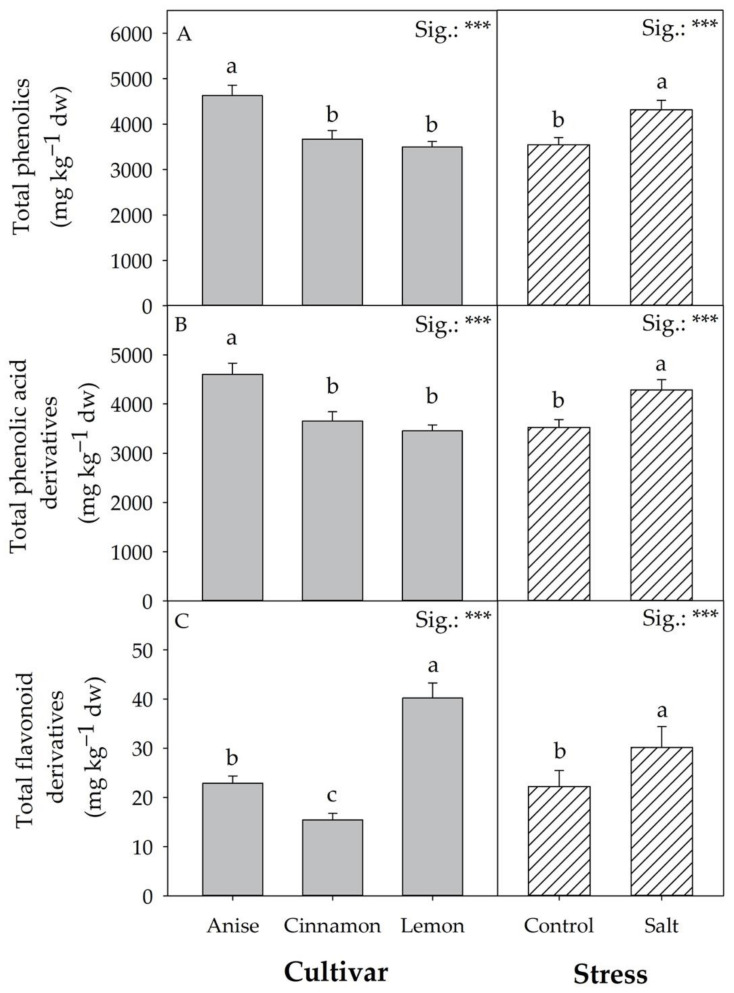
Effect of basil cultivars and stress on total phenolics (**A**), total phenolic acid derivatives (**B**), and total flavonoid derivatives (**C**). Data are mean values ± standard error, *n* = 3. Mean comparisons were performed by Tukey HSD test for CV and by *t*-Test for S. Different letters indicate significant mean differences. *** denotes significant effects at *p* ≤0.001.

As shown in Figure 6A, total phenolics were only affected by the average CV and S effects. Regardless of the stress condition, ‘Anise’ had the highest concentration of total phenolics (4625.09 mg kg^−1^ dw). Compared to the control, salt stress increased total phenolics by 21.63%.

Like total phenols, phenolic acid derivatives and total flavonoids showed a similar trend in response to salt stress (Figure 6B,C). Relative to the mean effect of the cultivar, ‘Anise’ showed the highest values of total phenolic acid derivatives (Figure 6B), in contrast to total flavonoid derivatives (‘Lemon’ > ’Anise’ > ’Cinnamon’; Figure 6C).

Compared to total flavonoid derivatives, rutin was not significantly affected by the interaction of the factors considered (CV × S), unlike what was observed for di-hydroquercetin glucoside and quercitin glucoside. Regarding these glycoside compounds, salt stress increased their concentrations in ‘Anise’ and ‘Cinnamon’, while no significant differences were observed in ‘Lemon’. In contrast, in ‘Lemon’, salt stress increased the concentration of quercetin glucoside (35.77 mg kg^−1^ dw; Table 4).

Regardless of the factors considered, cichoric acid was the predominant compound, followed by rosmarinic, feruloyl tartaric, salvianolic k, caffeic, salvianolic L, caftaric, salvianic A, chlorogenic, and salvianolic A acids. For chlorogenic, rosmarinic, and feruloyl tartaric acids, the CV × S interaction did not result in significant differences, unlike what was observed for the mean effects (CV and S). Specifically, salt stress, compared to the control, increased the concentrations of the above acids by 16.73, 21.28, and 31.22%, respectively. Relative to the mean effect of the cultivar, ‘Anise’ recorded the highest concentrations of cichoric and rosmarinic acids, while ‘Cinnamon’ had the highest content of feruloyl tartaric acid (Table 3).

Similarly to the findings for the above phenolic acids, the CV × S interaction was not significant for salvianolic K, salvianolic L, and chlorogenic acids. However, salt stress significantly increased the concentrations for the acids mentioned above compared to the control. Regardless of stress, ‘Anise’ was characterized by the highest concentration of salvianolic L and chlorogenic acids, contrary to what was observed for salvianolic K acid, which did not show a significant difference between cultivars (Table 3).

Unlike what was observed for the cichoric, rosmarinic, feruloyl tartaric, salvianolic K, caffeic, and salvianolic L acids, the concentrations of caftaric, salviananic acid A, chlorogenic, and salvianolic A acids were significantly affected by the CV × S interaction.

Salt stress increased salvianolinic acid A concentration in all cultivars, with the highest values obtained by Anise × Salt (16.68 mg kg^−1^ dw). The same interaction also resulted in the highest concentration of caftaric (81.68 mg kg^−1^ dw) and caffeic acids (140.57 mg kg^−1^ dw). In ‘Anise’ and ‘Cinnamon’, compared with the control, salt stress increased salvianic acid A concentrations by 32.36 and 15.50%, in contrast to ‘Lemon’, which did not show a significant difference.

### 3.6. Leaf Minerals

**Figure 7:**
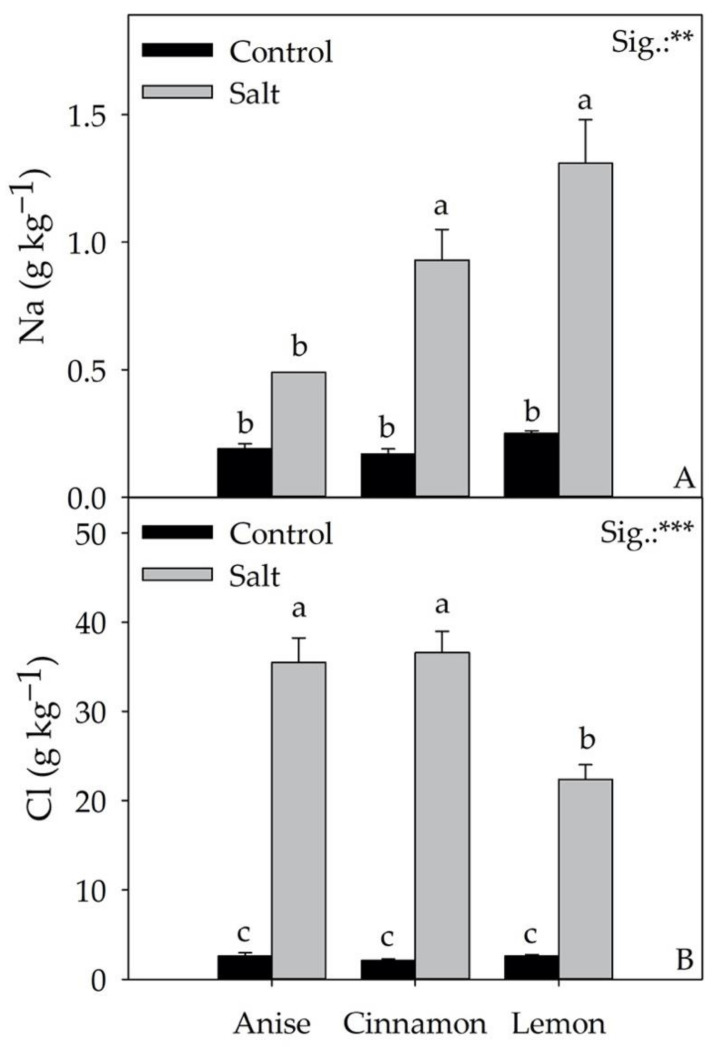
Effect of Cultivar × Stress on Na (**A**) and Cl (**B**) leaf concentration. Data are mean values ± standard error, *n* = 3. Statistical significance of the CV × S interaction was determined by Tukey HSD test for Cultivar and by *t*-Test for Stress. Different letters indicate significant mean differences. ** and *** denote non-significant or significant effects at *p* ≤ 0.01 and 0.001, respectively.

The effects of the factors considered (CV and S) and their interaction (CV × S) on the accumulation of minerals in basil leaves are shown in Table 5 and Figure 7. The concentrations of nitrate, Ca, Mg, Na, and Cl were influenced by the average CV and S effects and the CV × S interaction, except for K and P, where a significant difference was observed only for the average CV and S effects. Regardless of salt treatment, ‘Lemon’ had the highest P value (11.75 g kg^−1^ dw) while ‘Anise’ had an 8.7% higher K concentration than ‘Cinnamon’ (Table 5). Regardless of the cultivar, compared to the control, salt stress significantly reduced nitrate, K, and P concentrations (Table 5), while an opposite trend was observed for Cl (Figure 7). Compared to the CV × S interaction, the salt treatment cultivars ‘Cinnamon’ and ‘Lemon’ had significantly higher Na concentrations than the Control (Figure 7). The same treatment increased Ca and Mg concentrations in ‘Anise’ and ‘Cinnamon’ (Table 5).

## 4. Discussion

The significant difference recorded for total fresh weight in the basils evaluated in the present work underlines the strong impact of genotype. This variability was expected, assuming the phylogenetic gap between species and cultivars of the genus Ocimum. Regardless of salt treatment (Salt), ‘Cinnamon’ was the most productive cultivar, followed by ‘Anise’ and ‘Lemon’ (Figure 2). However, it should be noted that ‘Anise’ and ‘Cinnamon’ did not differ significantly in height, number of leaves, and leaf area, although the total fresh weight was different (Figure 3A–C). Relative to total fresh weight, the significant differences observed between cultivars did not result in a different response to salt stress, unlike what Ciriello et al. [27] observed in a recent work on basil. On average, salt treatment reduced the total fresh yield by 51.54% compared to the control; similar results were reported on *Salvia officinalis* L. [28] and *Mentha spicata* L. [29]. Regardless of cultivar, an adaptive response to salt stress was observed, culminating in reduced growth, an aspect that would allow plants to conserve energy by promoting the initiation of targeted defensive responses aimed at reducing permanent damage, as inferred from photosynthetic parameters shown in Table 2. Although Attia et al. [7] considered inappropriate to compare leaf production and photosynthetic activity (as it was related to a small number of leaves), in our study, salt stress reduced the net CO_2_ assimilation rate and transpiration. Like Bekhradi et al. [30] and Attia et al. [7], salt-induced osmotic and nutritional stress would prompt plants to reduce stomatal conductance, affecting RuBisCo activity. Although salt stress can cause severe damage to photosystems due to poor management of excess excitation energy, the plants’ morphophysiological and biochemical adaptations mitigated the deleterious effects induced by stress, as confirmed by F_v_/F_m_ values that never reached critical values (Figure 4D). However, it is essential to note that, although F_v_/F_m_ is the most widely used parameter for evaluating the performance of PSII under stress conditions [31], it may not be an acceptable parameter for assessing the physiological responses of plants under salt stress.

The analysis of morphological parameters of the salt-treated plants showed a lower sensitiveness of the Anise and Cinnamon cultivars than ‘Lemon’. The latter reduced leaf area and height by 57.38 and 35.37%, compared to the control; in contrast, ‘Anise’ and ‘Cinnamon’ reduced, on average, the same parameters by 33.28 and 19.87%. The above is confirmed by the reduction in total dry weight in ‘Lemon’ (56.97%) in the salt treatment compared to the control. In contrast, no significant changes were observed in ‘Cinnamon’ for this key parameter. This result could be partially attributable to a constitutive higher tolerance to salt stress and partially to the increase in total dry matter, which increased from 9.27% (Cinnamon × Control) to 15.10% (Cinnamon × Salt) (Figure 3E). As Bernstein et al. [32] suggested, good hydration of plant tissues is a key characteristic of salt stress tolerance. However, in the present study, the unchanged values of total dry weight in ‘Cinnamon’ cannot be considered indicators of salt stress tolerance.

It is important to emphasize that the reduction in fresh weight in all cultivars cannot be attributed solely to the expansion of the leaf area, which plays a key role in the regulation of the transpiration process under osmotic stress. According to several authors [7,33], the reduction in fresh weight is also attributable to a significant and general reduction in leaf initiation (number of leaves). However, the smaller leaf number and area reduction in the Cinnamon × Salt interaction compared to the Anise × Salt and Lemon × Salt ones would elect the Cinnamon cultivar as the most salt tolerant (Figure 3B,C). In our experiment, controlled salt stress induced in plants for 26 days resulted in ionic and nutritional stress, attributed to the accumulation of Na^+^ and Cl^−^ in transpiration fluxes, leading to a reduction in total fresh weight (Figure 2A and Figure 7).

Although Attia et al. [34] showed that, in basil, Na^+^ is partly transported by the xylem and accumulates in the roots, in our work, salt stress resulted in +355% Na^+^ accumulation regardless of the cultivar (Figure 7). This result does not exclude the defensive response of the plants to salt since Na^+^ values did not result in deleterious metabolic dysfunction. In general, the increase in dry matter percentage observed in all cultivars (+35.12%) would suggest that Na^+^ accumulation was not totally internalized in the leaves, a condition that, as reported by Attia et al. [7], would explain the changes in leaf water content. Although it is frequently documented in the literature [35] that high Na^+^ concentrations reduce K, Mg, and Ca uptake, only a reduction in K was observed in our experiment under salt stress; differentially, the concentration of Mg and Ca increased. As Scagel et al. [36] suggested, salt could have reduced Mg and Ca uptake while alternating the allocation of these elements, increasing their concentration. However, since Ca mediates different key processes of adaptation to stress conditions, including the Salt-Overly Sensitive (SOS) pathway, its increase could be related to a long-term response to salinity, as also argued by Mancarella et al. [5]. Similarly to what has been observed for Na^+^, the use of 60 mM NaCl in the nutrient solution increased the Cl concentration in the leaves, reaching an average value of 36 g kg^−1^ dw in the ‘Anise’ and ‘Cinnamon’, which explaining the observed worsening production performance (Figure 2 and Figure 3). In fact, the toxicity thresholds for salt-sensitive and salt-tolerant species are 7 and 50 g kg^−1^ dw, respectively [37]. As observed in green and red basil, the increase in Cl^−^ reduced the nitrate concentration (−77.21%), an antinutritional compound with a negative impact on human health. In salt treatment, the acknowledged antagonism between Cl^−^ and nitrate would have reduced the latter’s uptake, slowing the plants’ growth rate [38]. Although under salt stress, one of the most common physiological responses is chlorophyll degradation [30], regardless of the cultivar, we did not observe a reduction in chlorophyll a, b, or total chlorophyll concentrations (Table 2). Consistent with the observations of Bernstein et al. [32], this result suggests that this parameter should be considered an indicator of salt stress for basil. Partially in agreement with the above, the main colorimetric parameters in ‘Anise’ and ‘Cinnamon’ under salt stress did not change significantly from what was recorded in the Control (Table 1), considering that leaf color is often correlated with chlorophyll concentration [32]. However, for these two cultivars, salt stress did not significantly alter L, b, chroma, hue angle and especially a*, referred to in the literature as greenness; these results are probably attributable to the non-significant change in chlorophyll concentration under salt stress.

Unlike in ‘Lemon’, salt significantly altered colorimetric parameters (Table 1) compared to chlorophyll, and this result could be attributed to significant increases in lutein and β-carotene concentration (Table 2). In addition to their central role as supplementary pigments in photosynthetic processes, carotenoids are crucial for photoprotection due to their antioxidant properties [39]. As Bernstein et al. [32] hypothesized, an increase in carotenoids under salt stress would indicate a protective mechanism against stress, as it is universally recognized that environmental signals control the genetic regulation involved in their biosynthesis and bioaccumulation. Similarly to what was observed for carotenoids, salt stress, regardless of cultivar, increased the concentration of flavonoid derivatives (+35.17) and phenolic acids (+21.54%) and therefore total phenolic compounds (+21.63%; Figure 6). This result is not unexpected, as under NaCl salt stress, the energy stored during photosynthesis is used mainly for growth, with only a part used for synthesizing secondary metabolites. However, mainly by limiting growth, salt would prompt plants to allocate more energy to produce low-carbon secondary metabolites (such as polyphenols) that would help combat ROS [40]. Ghorbanpour and Varma [35] state that the increase in polyphenol concentration in response to salt is related to specific enzymes, including PAL (phenylalanine ammonia lyase). The drastic reduction in osmotic potential, a consequence of salt stress, increases L-proline levels, which is primarily responsible for the osmotic rebalancing. Although this amino acid is a primary metabolite, it may be related to the production of phenolic compounds due to its relationship with PAL [41]. Regardless of salt stress, it is worth noting again how the choice of genetic material significantly affects the bioaccumulation of phenolic compounds [17,42,43]. ‘Anise’ showed an average concentration of total phenolic compounds 29.12% higher than that recorded in ‘Lemon’ and ‘Cinnamon’. Despite this, the most abundant phenolic acid was chicoric acid, which on average accounted for 63.51% of the phenolic acid derivatives. Although rosmarinic acid is listed in the literature as the most abundant phenolic acid in basil [44], a study of 15 basil cultivars confirmed the influence of genotype on both the quantity and quality of the phenolic profile [45].

‘Lemon’ was characterized by a 160.57% higher total concentration of flavonoid derivatives compared with ‘Cinnamon’. This remarkable difference partially justifies the significant colorimetric differences observed between cultivars, as flavonoids also confer color to vegetables [46]; what has been observed may help to understand the results related to antioxidant activity (Figure 5). Regardless of the methodology used to determine antioxidant activity (DPPH, ABTS, and FRAP), salt increased antioxidant activity, probably due to increased production of compounds with antioxidant activity (carotenoids, phenolic acid derivatives, and flavonoids) [4]. It is well known that phenolic compounds contribute to the antioxidant activity of plant matrices. Therefore, according to Kwee and Niemeyer [45], the different phenolic profile of the three basil cultivars affected the antioxidant power and the activity of DPPH, ABTS, and FRAP (Figure 5). With a 29.50% (on average) concentration of phenolic acid derivatives higher than the other cultivars, ‘Anise’ showed the highest level of antioxidant activity. On the contrary, although ‘Cinnamon’ constitutively had a higher concentration of carotenoid (lutein and *β*-carotene) than ‘Lemon’, it had lower mean values of DPPH, ABTS, and FRAP. This result could be attributable to the higher constitutive concentration of flavonoids in ‘Lemon’, which by structural characteristics may have positively influenced the antioxidant activities of DPPH, ABTS, and FRAP.

## 5. Conclusions

**Figure 8:**
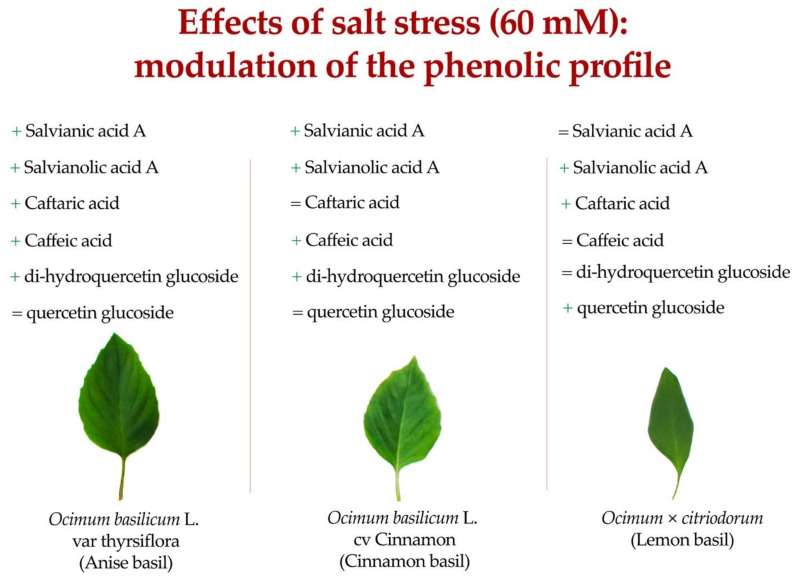
Effect of salt stress on representative phenolic compound of ‘Anise’, ‘Cinnamon’, and ‘Lemon’ basil.

Despite the phylogenetic distance and significant differences in yield and quality, the three basil types (‘Anise’, ‘Cinnamon’, and ‘Lemon’) tested had similar responses to NaCl salinization. Specifically, salinized plants enacted adaptive stress responses mainly based on reduced photosynthetic activity and growth (total fresh weight, fresh leaf weight, height, number of leaves, leaf area, and total dry weight) but at the same time increased secondary metabolites, increasing their antioxidant properties. Specifically, as shown in Figure 8, salt stress modulated the production of polyphenolic compounds in a cultivar-dependent manner. Although the moderate tolerance of basil to salinity is noted several times, we can state that the use of 60 mM NaCl in the nutrient solution is limiting for the production of the basil tested. In any case, considering the interest of the cosmetic, pharmaceutical and perfume industries in secondary metabolites with bioactive action, the feasibility of using salt stress as an enhancer of such compounds could be explored.

## Figures and Tables

**Table 1:** Effect of basil cultivars (CV) and stress (S) on CIELab colorimetric indices.

Treatment	L	a	b	Chroma	Hue Angle
**Cultivar (CV)**					
Anise	42.45 ± 0.26 b	–12.61 ± 0.43 a	17.54 ± 0.62 b	21.61 ± 0.75 b	125.76 ± 0.25
Cinnamon	42.84 ± 0.54 b	–13.03 ± 0.30 a	18.14 ± 0.78 b	22.35 ± 0.86 b	125.85 ± 0.62
Lemon	44.10 ± 0.36 a	–14.77 ± 0.68 b	20.46 ± 1.42 a	25.25 ± 1.55 a	126.03 ± 0.65
**Stress (S)**					
Control	43.83 ± 0.33	–14.37 ± 0.53	20.54 ± 0.88	25.08 ± 1.04	125.07 ± 0.28
Salt	42.43 ± 0.31	–12.57 ± 0.21	16.89 ± 0.20	21.06 ± 0.26	126.70 ± 0.35
**CV × S**					
Anise × Control	42.90 ± 0.29	–13.33 ± 0.61 ab	18.46 ± 0.92 b	22.77 ± 1.10 b	125.89 ± 0.31 ab
Anise × Salt	42.00 ± 0.23	–11.90 ± 0.21 a	16.63 ± 0.48 b	20.46 ± 0.50 b	125.64 ± 0.45 b
Cinnamon × Control	43.80 ± 0.57	–13.52 ± 0.47 b	19.57 ± 0.96 b	23.79 ± 1.27 b	124.66 ± 0.41 b
Cinnamon × Salt	41.88 ± 0.45	–12.55 ± 0.06 ab	16.71 ± 0.23 b	20.90 ± 0.15 b	127.04 ± 0.57 a
Lemon × Control	44.79 ± 0.09	–16.27 ± 0.17 c	23.59 ± 0.58 a	28.66 ± 0.58 a	124.65 ± 0.38 b
Lemon × Salt	43.41 ± 0.40	–13.27 ± 0.16 ab	17.34 ± 0.16 b	21.84 ± 0.21 b	127.41 ± 0.17 a
**Significance**					
CV	**	***	***	***	ns
S	***	***	***	***	***
CV × S	ns	*	*	*	**

Data are mean values ± standard error, *n* = 3. Mean comparisons were performed by Tukey HSD test for CV and by *t*-Test for S. Different letters within each column indicate significant mean differences. ns, *, **, and *** denote non-significant or significant effects at *p* ≤ 0.05, 0.01, and 0.001, respectively.

**Table 2:** Effect of basil cultivars (CV) and stress (S) on Chlorophylls and carotenoids.

Treatment	Chlorophyll a	Chlorophyll b	Total Chlorophyll	Lutein	*β*-Carotene
	mg g^−1^ fw			mg kg^−1^ fw	
**Cultivar (CV)**					
Anise	1.21 ± 0.01	0.80 ± 0.03	2.01 ± 0.04	1305.20 ± 75.22 a	556.82 ± 40.88a
Cinnamon	1.22 ± 0.01	0.79 ± 0.01	2.01 ± 0.02	1243.47 ± 92.75 a	555.45 ± 47.88 a
Lemon	1.21 ± 0.01	0.89 ± 0.05	2.10 ± 0.05	953.05 ±6 3.54 b	511.73 ± 4.51 b
**Stress (S)**					
Control	1.22 ± 0.01	0.86 ± 0.04	2.08 ± 0.04	998.24 ± 50.62	478.50 ± 11.22
Salt	1.21 ± 0.01	0.79 ± 0.02	2.00 ± 0.02	1336.24 ± 61.77	604.17 ± 25.62
**CV × S**					
Anise × Control	1.23 ± 0.00	0.84 ± 0.05	2.07 ± 0.05	1139.44 ± 22.43	465.63 ± 5.39 d
Anise × Salt	1.19 ± 0.01	0.75 ± 0.03	1.95 ± 0.03	1470.97 ± 17.47	648.02 ± 2.88 a
Cinnamon × Control	1.20 ± 0.02	0.79 ± 0.01	1.99 ± 0.03	1042.12 ± 41.09	448.53 ± 5.11 e
Cinnamon × Salt	1.24 ± 0.01	0.80 ± 0.03	2.04 ± 0.03	1444.81 ± 28.04	662.38 ± 1.61 a
Lemon × Control	1.22 ± 0.01	0.96 ± 0.07	2.17 ± 0.08	813.16 ± 22.23	521.35 ± 2.27 b
Lemon × Salt	1.20 ± 0.01	0.82 ± 0.03	2.02 ± 0.03	1092.94 ± 11.14	502.11 ± 1.97 c
**Significance**					
CV	ns	ns	ns	***	***
S	ns	ns	ns	***	***
CV × S	ns	ns	ns	ns	***

Data are mean values ± standard error, *n* = 3. Mean comparisons were performed by Tukey HSD test for CV and by *t*-Test for S. Different letters within each column indicate significant mean differences. ns and *** denote non-significant or significant effects at *p* ≤ 0.001, respectively.

**Table 3:** Effect of basil cultivars (CV) and stress (S) on phenolic acid derivatives.

Treatment	Salvianic Acid A	Salvianolic Acid k	Salvianolic Acid A	Salvianolic Acid L	Caftaric Acid	Rosmarinic Acid	Cichoric Acid	Caffeic Acid	Chlorogenic Acid	Feruloyl Tartaric Acid
	mg kg^−1^ dw									
**Cultivar (CV)**										
Anise	34.25 ± 2.34 b	100.50 ± 7.14	15.16 ± 0.69 a	75.18 ± 2.66 a	72.26 ± 4.48 a	1045.78 ± 51.34 a	2867.13 ± 143.05 a	128.18 ± 5.82 a	29.10 ± 1.13 a	234.67 ± 17.55 b
Cinnamon	39.53 ± 1.35 a	107.78 ± 6.93	2.73 ± 0.30 c	35.69 ± 2.26 c	21.28 ± 1.59 c	689.18 ± 35.78 c	2365.51 ± 125.86 b	45.78 ± 2.5 b	11.99 ± 0.82 c	334.54 ± 16.14 a
Lemon	6.00 ± 0.40 c	96.47 ± 6.87	7.02 ± 0.56 b	47.88 ± 2.87 b	28.30 ± 2.87 b	882.79 ± 33.32 b	2204.55 ± 62.98 b	48.03 ± 1.34 b	18.26 ± 1.08 b	114.86 ± 10.28 c
**Stress (S)**										
Control	23.77 ± 4.80	87.45 ± 2.48	7.19 ± 1.70	47.66 ± 5.89	34.38 ± 7.15	788.65 ± 49.42	2252.68 ± 90.84	67.13 ± 12.21	17.73 ± 2.47	197.23 ± 30.45
Salt	29.42 ± 5.68	115.71 ± 3.22	9.43 ± 1.95	58.18 ± 5.91	46.85 ± 8.90	956.51 ± 56.59	2705.44 ± 124.97	80.86 ± 14.96	21.84 ± 2.58	258.81 ± 33.66
**CV × S**										
Anise × Control	29.48 ± 1.76 c	86.79 ± 4.41	13.65 ± 0.15 b	69.97 ± 2.74	62.83 ± 0.77 b	944.97 ± 32.94	2589.89 ± 97.13	115.79 ± 2.16 b	26.94 ± 1.06	198.92 ± 15.52
Anise × Salt	39.02 ± 1.20 ab	114.21 ± 6.91	16.68 ± 0.31 a	80.40 ± 0.73	81.68 ± 3.34 a	1146.59 ± 43.95	3144.36 ± 126.56	140.57 ± 3.37 a	31.26 ± 0.77	270.41 ± 4.69
Cinnamon × Control	36.69 ± 0.36 b	93.65 ± 3.41	2.11 ± 0.19 f	31.09 ± 1.27	18.33 ± 1.25 d	611.74 ± 8.13	2089.00 ± 28.07	40.38 ± 0.92 d	10.34 ± 0.71	300.37 ± 7.99
Cinnamon × Salt	42.38 ± 0.94 a	121.91 ± 5.34	3.36 ± 0.18 e	40.29 ± 1.64	24.23 ± 1.57 d	766.62 ± 18.34	2642.02 ± 44.20	51.18 ± 1.09 c	13.64 ± 0.34	368.71 ± 8.49
Lemon × Control	5.13 ± 0.10 d	81.93 ± 2.87	5.80 ± 0.11 d	41.92 ± 2.31	21.96 ± 0.19 d	809.26 ± 8.96	2079.15 ± 59.19	45.23 ± 0.93 cd	15.90 ± 0.19	92.41 ± 2.54
Lemon × Salt	6.87 ± 0.22 d	111.01 ± 4.08	8.25 ± 0.21 c	53.84 ± 0.61	34.64 ± 0.93 c	956.32 ± 7.97	2329.94 ± 24.67	50.83 ± 0.54 c	20.62 ± 0.44	137.31 ± 4.20
**Significance**										
CV	***	ns	***	***	***	***	***	***	***	***
S	***	***	***	***	***	***	***	***	***	***
CV × S	**	ns	**	ns	**	ns	ns	***	ns	ns

Data are mean values ± standard error, *n* = 3. Mean comparisons were performed by Tukey HSD test for CV and by *t*-Test for S. Different letters within each column indicate significant mean differences. ns, **, and *** denote non-significant or significant effects at *p* ≤ 0.01 and 0.001, respectively.

**Table 4:** Effect of basil cultivars (CV) and stress (S) on flavonoids derivatives.

Treatment	Di-Hydroquercetin Glucoside	Rutin	Quercetin Glucoside
	mg kg^−1^ dw		
**Cultivar (CV)**			
Anise	3.66 ± 0.18 a	9.32 ± 0.78 b	9.91 ± 0.54 b
Cinnamon	0.87 ± 0.09 b	10.77 ± 0.79 a	3.78 ± 0.49 c
Lemon	0.52 ± 0.02 c	9.08 ± 0.41 b	30.59 ± 2.67 a
**Stress (S)**			
Control	1.48 ± 0.45	8.36 ± 0.26	12.33 ± 3.47
Salt	1.89 ± 0.54	11.09 ± 0.43	17.18 ± 4.75
**CV × S**			
Anise × Control	3.29 ± 0.06 b	7.69 ± 0.25	8.86 ± 0.54 cd
Anise × Salt	4.04 ± 0.10 a	10.95 ± 0.58	10.95 ± 0.25 c
Cinnamon × Control	0.68 ± 0.02 d	9.15 ± 0.40	2.74 ± 0.28 e
Cinnamon × Salt	1.06 ± 0.01 c	12.39 ± 0.56	4.82 ± 0.22 de
Lemon × Control	0.48 ± 0.03 d	8.23 ± 0.26	25.40 ± 2.62 b
Lemon × Salt	0.56 ± 0.01 d	9.92 ± 0.20	35.77 ± 1.40 a
	**Significance**		
CV	***	**	***
S	***	***	***
CV × S	***	ns	**

Data are mean values ± standard error, *n* = 3. Mean comparisons were performed by Tukey HSD test for CV and by *t*-Test for S. Different letters within each column indicate significant mean differences. ns, **, and *** denote non-significant or significant effects at *p* ≤ 0.01 and 0.001, respectively.

**Table 5:** Effect of basil cultivars (CV) and stress (S) on Nitrate, K, P, Ca, and Mg leaf concentration.

Treatment	Nitrate	K	P	Ca	Mg
	mg g^−1^ fw	g kg^−1^ dw			
**Cultivar (CV)**					
Anise	2555.76 ± 785.29 a	38.25 ± 3.24 a	6.83 ± 0.43 b	9.19 ± 1.04 ab	5.97 ± 0.71 a
Cinnamon	1542.67 ± 361.53 b	31.72 ± 1.34 b	6.35 ± 0.40 b	9.32 ± 1.00 a	5.73 ± 0.84 a
Lemon	1403.80 ± 411.30 b	35.16 ± 2.59 ab	11.75 ± 0.45 a	7.53 ± 0.27 b	4.37 ± 0.14 b
**Stress (S)**					
Control	2987.44 ± 332.92	39.26 ± 1.95	8.99 ± 0.92	7.29 ± 0.26	4.26 ± 0.15
Salt	680.71 ± 66.37	30.82 ± 1.20	7.64 ± 0.87	10.07 ± 0.71	6.46 ± 0.56
**CV × S**					
Anise × Control	4303.80 ± 136.53 a	44.01 ± 3.76	7.24 ± 0.66	7.33 ± 0.67 b	4.51 ± 0.3 b
Anise × Salt	807.72 ± 95.40 c	32.49 ± 2.25	6.42 ± 0.59	11.05 ± 1.21 a	7.44 ± 0.51 a
Cinnamon × Control	2341.87 ± 39.22 b	33.24 ± 0.52	7.16 ± 0.39	7.18 ± 0.50 b	3.90 ± 0.24 b
Cinnamon × Salt	743.47 ± 115.19 c	30.19 ± 2.53	5.55 ± 0.12	11.47 ± 0.38 a	7.57 ± 0.32 a
Lemon × Control	2316.65 ± 100.39 b	40.54 ± 0.98	12.56 ± 0.34	7.36 ± 0.28 b	4.37 ± 0.11 b
Lemon × Salt	490.94 ± 49.49 c	29.78 ± 1.93	10.94 ± 0.51	7.70 ± 0.50 b	4.37 ± 0.29 b
	**Significance**				
CV	***	*	***	*	***
S	***	***	**	***	***
CV × S	***	ns	ns	*	***

Data are mean values ± standard error, *n* = 3. Mean comparisons were performed by Tukey HSD test for CV and by *t*-Test for S. Different letters within each column indicate significant mean differences. ns, *, **, and *** denote non-significant or significant effects at *p* ≤ 0.05, 0.01 and 0.001, respectively.

## Data Availability

The datasets generated for this study are available on request to the corresponding author.

## Supplementary Materials

The following supporting information can be downloaded at: https://www.mdpi.com/article/10.3390/antiox11112207/s1, Table S1: Effect of basil cultivars (CV) and stress (S) on plant height, leaf number, leaf area, total dry weight, and total dry matter; Table S2: Effect of basil cultivars (CV) and stress (S) on leaf transpiration (E), leaf stomatal conductance (gs), CO_2_ net assimilation rate, and Chlorophyll fluorescence (Fv/Fm).

## Abbreviations

NS	nutrient solution
ACO_2_	net assimilation rate of CO_2_
E	transpiration
Gs	stomatal conductance
HPLC-DAD	high-performance liquid chromatography with diode array detection
DPPH	(1,1-diphenyl-2-picrylhydrazyl)
ABTS	(2,2′-azinobis-(3-ethylbenzothiazoline-6-sulfonate)
FRAP	(ferric ion reducing antioxidant power)
UHPLC	ultrahigh performance liquid chromatography
HRMS	high resolution mass spectrometry
