# Supplementary material for: Morpho-Physiological and Biochemical Responses of Hydroponically Grown Basil Cultivars to Salt Stress
Source: Antioxidants (Basel). 2022 Nov 8;11(11):2207. doi: 10.3390/antiox11112207 (PMC9686911; doi:10.3390/antiox11112207)
Supplement: Supplementary file 1 [file antioxidants-11-02207-s001.zip › antioxidants-1988751-supplementary.pdf]

antioxidants

MDPI

Supplementary Table S1. Effect of basil cultivars (CV) and stress (S) on plant height, leaf number, leaf area, total dry weight, and total dry matter.

Treatment	Plant height	Leaf number	Leaf area	Total dry weight	Total Dry matter
	cm	n. plant^{-1}	cm^2	g plant^{-1}	%
**Cultivar (CV)**					
Anise	46.04 ± 2.53 a	233.22 ± 25.97 a	1587.66 ± 184.58 a	15.33 ± 1.52 a	13.24 ± 0.80
Cinnamon	46.53 ± 2.16 a	235.47 ± 10.01 a	1473.43 ± 99.68 a	14.89 ± 0.42 a	12.19 ± 1.33
Lemon	42.39 ± 4.09 b	209.14 ± 26.93 b	1213.58 ± 218.65 b	10.31 ± 1.86 b	13.07 ± 0.63
**Stress (S)**					
Control	51.43 ± 0.47	271.72 ± 5.85	1791.55 ± 54.93	16.19 ± 0.68	10.92 ± 0.45
Salt	38.54 ± 1.36	180.17 ± 10.12	1058.23 ± 88.05	10.83 ± 1.23	14.75 ± 0.32
**CV × S**					
Anise × Control	51.56 ± 1.11 a	290.00 ± 7.09 a	1991.39 ±7 3.53 a	18.63 ± 0.49 a	11.62 ± 0.2 b
Anise × Salt	40.52 ± 0.56 b	176.44 ± 9.97 d	1183.93 ± 44.02 c	12.03 ± 0.67 c	14.86 ± 0.76 a
Cinnamon × Control	51.22 ± 0.81 a	256.11 ± 7.56 b	1681.39 ± 18.71 b	15.53 ± 0.63 b	9.27 ± 0.45 c
Cinnamon × Salt	41.83 ± 0.79 b	214.83 ± 4.26 c	1265.47 ± 77.97 c	14.26 ± 0.28 bc	15.10 ± 0.32 a
Lemon × Control	51.50 ± 0.87 a	269.06 ± 3.31 ab	1701.87 ± 19.71 b	14.41 ± 0.41 bc	11.86 ± 0.33 b
Lemon × Salt	33.28 ± 0.15 c	149.22 ± 4.89 d	725.29 ± 14.65 d	6.20 ± 0.47 d	14.29 ± 0.62 a
**Significance**					
CV	***	**	***	***	ns
S	***	***	***	***	***
CV × S	***	***	***	***	*

Data are mean values ± standard error, $n = 3$. Mean comparisons were performed by Tukey HSD test for CV and by *t*-Test for S. Different letters within each columns indicate significant mean differences. ns, *, **, and *** denote non-significant or significant effects at $p \leq 0.05$, 0.01, and 0.001, respectively.

ì

Supplementary Table S2. Effect of basil cultivars (CV) and stress (S) on leaf transpiration (E), leaf stomatal conductance (gs), CO_2 net assimilation rate, and Chlorophyll fluorescence (Fv/Fm).

Treatment	E<br>mol H_2O m^{-2} s^{-1}	gs<br>mol H_2O m^{-2} s^{-1}	ACO_2<br>μmol CO_2 m^{-2} s^{-1}	Fv/Fm
**Cultivar (CV)**				
Anise	5.11 ± 0.41 b	0.18 ± 0.02 b	15.34 ± 0.75 ab	0.78 ± 0.01
Cinnamon	5.64 ± 0.65 ab	0.20 ± 0.02 ab	16.81 ± 1.11 a	0.77 ± 0.01
Lemon	5.97 ± 0.41 a	0.24 ± 0.02 a	15.11 ± 1.23 b	0.77 ± 0.02
**Stress (S)**				
Control	6.59 ± 0.20	0.24 ± 0.01	17.87 ± 0.50	0.79 ± 0.00
Salt	4.55 ± 0.22	0.17 ± 0.02	13.63 ± 0.39	0.76 ± 0.01
**CV × S**				
Anise × Control	6.02 ± 0.13	0.23 ± 0.01	16.81 ± 0.63	0.80 ± 0.01
Anise × Salt	4.20 ± 0.10	0.13 ± 0.01	13.87 ± 0.52	0.77 ± 0.01
Cinnamon × Control	7.05 ± 0.24	0.24 ± 0.02	19.20 ± 0.52	0.79 ± 0.00
Cinnamon × Salt	4.23 ± 0.27	0.15 ± 0.01	14.42 ± 0.42	0.76 ± 0.01
Lemon × Control	6.71 ± 0.36	0.25 ± 0.01	17.61 ± 0.91	0.79 ± 0.01
Lemon × Salt	5.23 ± 0.39	0.22 ± 0.04	12.61 ± 0.70	0.75 ± 0.03
		**Significance**		
CV	*	**	*	ns
S	***	***	***	*
CV × S	ns	ns	ns	ns

Data are mean values ± standard error, n = 3. Mean comparisons were performed by Tukey HSD test for CV and by *t*-Test for S. Different letters within each columns indicate significant mean differences. ns, *, **, and *** denote non-significant or significant effects at $p \leq 0.05$, 0.01, and 0.001, respectively.
